# Income in Relation to Psychosocial Factors Among Stroke Survivors using Smartwatches for Atrial Fibrillation Monitoring

**DOI:** 10.26502/fccm.92920404

**Published:** 2024-10-11

**Authors:** Syed Naeem, Tyler Jones, Joseph Daniel, Jordy Mehawej, Andreas Filippaios, Tenes Paul, Ziyue Wang, Sakeina Howard-Wilson, Darleen Lessard, Eric Ding, Edith Mensah Otabil, Kamran Noorishirazi, Apurv Soni, Jane Saczynski, Khanh-Van Tran, David McManus

**Affiliations:** a Department of Medicine, University of Massachusetts Chan Medical School, 55 Lake Avenue North, Worcester, MA, USA; b Department of Population and Quantitative Health Sciences, University of Massachusetts Medical School, 55 Lake Avenue North, Worcester, MA, USA; c Department of Pharmacy and Health Systems Sciences, School of Pharmacy, Northeastern University, 360 Huntington Ave, Boston, MA, USA

**Keywords:** Atrial Fibrillation (AF), Socioeconomic Status (SES), Generalized Anxiety Disorder-7 Scale (GAD-7), Consumer Health Activation Index (CHAI), Short Form Survey (SF-12), Physical Component Score (PCS), Mental Component Score (MCS)

## Abstract

**Background:**

Timely detection of atrial fibrillation (AF) is critical for stroke prevention. Smartwatches are FDA-approved devices that can now aide in this detection.

**Objective:**

Investigate how socioeconomic status is associated with self-reported psychosocial outcomes, including anxiety, patient activation, and health-related quality of life in stroke survivors using smartwatch for AF detection.

**Methods:**

We analyzed data from the Pulsewatch study, a randomized controlled trial (NCT03761394). Participants in the intervention group wore a cardiac patch monitor in addition to a smartwatch for AF detection, whereas the control group wore only the cardiac patch monitor. Generalized anxiety disorder-7 scale, Consumer Health Activation Index and short-form health survey were completed to assess anxiety, patient activation, physical and mental health status at baseline, 14, and 44 days. We used a longitudinal linear regression model to examine changes in psychosocial outcomes in low (<$50K) vs. high (>$50K) income groups.

**Results:**

A total of 95 participants (average age 64.9± 9.1 years; 57.9% male; 89.5% non-Hispanic white) were included. History of renal disease (p-value 0.029), statin use (p-value 0.034), depression (p-value 0.004), and anxiety (p-value <0.001), were different between the income groups. In the adjusted model, the low-income group was associated with increased anxiety (β 2.75, p-value 0.0003), and decreased physical health status (β −5.07, p-value 0.02). There was no change identified in self-reported patient engagement and mental health status score.

**Conclusion:**

Our findings demonstrate that low SES is associated with worse self-reporting of physical health status, and this may influence psychosocial outcomes in smartwatch users.

## 1. Introduction

Atrial Fibrillation (AF) is the most common cardiac arrhythmia, with a two-to-five-fold higher risk of stroke, many of which are preventable, if detected early [1]. Smartwatches have come to the forefront recently in detection of AF as their utilization has rapidly increased worldwide [2,3]. Currently more than five billion people own mobile phones, expanding the ability to deliver healthcare digitally [4]. However, smartwatches are expensive consumer products, and socioeconomic status (SES) and demographic differences exist in adopting this wearable device [2]. Therefore, these devices are primarily purchased and used by affluent young adults [3]. There is an increased risk of atrial fibrillation among those with lower incomes at an earlier age [5]. The prevalence of smartwatches among wealthier adults, which can screen for atrial fibrillation, might lead to a disparity in diagnosing and treating this condition, potentially leaving those in lower SES groups at a disadvantage [4].

Prior studies concerning SES have examined interaction with patient outcomes, including anxiety and physical and mental well-being [1]. Evidence reveals that low-SES individuals are disinclined to participate in shared medical decision-making, and as a result demonstrate less patient engagement [6]. Consequently, smartwatch usage in low-SES could be a barrier in the detection of AF especially in post-stroke patients. Motivated consumers with high SES and healthier lifestyles will likely purchase smartwatches, while low-SES individuals are less likely to do so [4,7]. However, the implementation of smartwatches for AF detection is becoming more successful and this poses an opportunity for lower SES individuals with AF to have unprecedented access to digital health tools like never before [2]. Generally wearable usage is a positive experience for users, with the devices being a source of multiple psychological benefits and few negative psychological implications [8]. In contrast, there are claims that wearable usage leads to negative experiences and feelings of anxiety and guilt [9]. This negative effect is relatively uncommon, but is more likely amongst individuals who simply do not wear their device [10]. Several studies have demonstrated that low-SES individuals tend also to have lower eHealth literacy and consequently do not incur the same benefits as their higher SES counterparts when engaging with digital health technologies, including smartwatches [11]. Nonetheless, increased access to smartwatches may result in increased participation and therefore, increased AF detection [12].

The relationship between socioeconomic status with anxiety, patient activation, and physical and mental health has not yet been explored in post-stroke survivors using smartwatches for AF monitoring. Therefore, using data from the Pulsewatch study, which enrolled stroke survivors using smartwatch monitoring for AF, we aim to evaluate how socioeconomic status is associated with anxiety, patient activation, and self-reported physical and mental health.

## 2. Methods

### 2.1 Study design and population

We used data from the Pulsewatch study, a randomized clinical trial designed to assess the accuracy and impact of a smartwatch prescribed for AF detection in stroke survivors. The intervention arm received the Pulsewatch system, which comprised of a smartwatch synced with a smartphone application that could detect AF. The control group received an ECG patch monitor, the standard of care with no additional devices. The protocol for the multiphase Pulsewatch study has been previously described [13]. The Institutional Review Board approved all study protocols at the University of Massachusetts Chan Medical School. Participants were considered eligible to participate if they: (1) were aged 50 years or more, (2) had a history of stroke or transient ischemic attack (TIA), (3) had a CHA2DS2Vasc risk score ≥ 2, (4) had no contraindication to anticoagulation, (5) presented at the in-patient service or ambulatory clinic (neurology and cardiovascular clinics), (6) could provide informed consent, and (7) were willing and capable of using the Pulsewatch system (smartwatch and smartphone app) daily to examine the accuracy and usability of a smartphone/smartwatch for AF detection over 44 days [13].

Participants were excluded from participation if they (1) had a significant contraindication to anticoagulation treatment (e.g., major hemorrhagic stroke); (2) had plans to move out of the area over the 44-day follow-up period; (3) were unable to read or speak the English language; (4) were unable to provide informed consent; (5) had a known allergy or hypersensitivity to medical-grade hydrocolloid adhesives or hydrogel; (6) had a life-threatening arrhythmia that required in-patient monitoring for immediate analysis; and (7) had an implantable pacemaker, as paced beats interfere with the ECG reading [13].

### 2.2 Study procedures

Participants provided clinical history and answered questions concerning their overall health status at the start of the study. Study research coordinators collected information regarding socio-demographics, past medical history, and medication use from the electronic medical record. Participants completed validated questionnaires, including the generalized anxiety disorder-7 (GAD-7) scale to determine the level of anxiety, the consumer health activation index to assess the level of health engagement, and the short form survey (SF-12) to determine the perception of their physical and mental health at enrollment, baseline, day-14, and day-44 on follow-up visits. Socioeconomic status was assessed by collecting education level information and total annual household income before taxes [13].

### 2.3 Socioeconomic status

All participants provided information on their annual household income before taxes. The Pulsewatch database divided the annual household income into eight categories: <$10000, $10000-$19999, $20000-$34999, $35000-$49999, $50000-$74999, $75000-$99999, $100000-$149999 and ≥$150000. For this analysis, we combined the eight categories into two groups: participants with <$50000 in the low-income group and participants with ≥$50000 in the high-income group.

### 2.4 Patient Reported Outcomes

Anxiety was assessed using the Generalized anxiety disorder scale (GAD-7), a validated instrument. Scores ranged from 0 to 27, with scores of 5, 10, and 15 representing validated cut-points for mild, moderate, and severe anxiety symptoms, respectively. A score of ≥ 5 was classified as the presence of anxiety [14,15].

The Consumer Health Activation Index (CHAI), a 10-item scale, was used to assess patient activation (referring to their ability and willingness to manage their health). Scores ranged from 10 to 60, then transformed via a linear transformation to a scale from 0 to 100, with a higher score associated with fewer depressive and anxiety symptoms and more excellent physical functioning [13].

We used the validated Short Form SF-12 questionnaire with Physical Component Score (PCS) and Mental Component Score (MCS) to assess health-related quality of life. Scores range from 0 to 100, with higher scores representing a better quality of life compared to the average [16].

We compared the baseline socio-demographic and clinical characteristics of participants in the high-income group to those in the low-income group. All categorical variables are represented as frequencies, and continuous variables are represented as means. Groups were compared using Pearson’s chi-square test for categorical data and an independent two-sample t-test for continuous data.

We then used a longitudinal linear regression model to examine participant factors that were associated with low- and high-income groups and changes in anxiety, patient activation, and self-reported physical and mental health. Model building was performed by adjusting for confounding variables based on whether they varied significantly between the income groups in the univariate models and based on their clinical relevance. To examine if there was any association between anxiety and the low-income group, we adjusted for baseline significant variables, including the history of renal disease and statin medication. To assess the association of the low-income group with CHAI score and SF-12 (PSC/MCS), we adjusted for baseline anxiety, history of renal disease, and statin use. Analyses were statistically significant if two-tailed P values were < 0.05. All statistical analyses were performed using SAS version 9.4 (SAS Institute, Inc., Cary, North Carolina, USA).

## 3. Results

A total of 95 participants with an average age of 64.9 ± 9.1 years were enrolled in the intervention arm of the Pulsewatch study, which was further stratified into two socio-economic groups: a low-income group (n=33, 34.7%) and a high-income group (n=62, 65.3%). Overall, the majority were male (55 %) and non-Hispanic white (90%) (Table 1). At baseline, participants with renal disease (12.1% vs 1.6%, p-value 0.029), depression (65.6% vs 35.5%, p-value 0.004), and anxiety (54.5% vs 18.3%, p-value <0.001) were more likely to be in the low-income group and participants with a history of statin use (84.9% vs.96.8%, p-value 0.034) were more likely to be in the high-income group (Table 1). Of note, wear time between the two income groups was not significantly different (Table 1, 3.77 vs 4.16 hours, p-value 0.45).

After 44 days of smartwatch prescription for AF monitoring, participants in the low-income group who were prescribed smartwatches for AF detection had reported increased anxiety and reduced self-rated physical health compared to participants in the high-income group (Table 2; β 2.75, p-value <0.001; β −5.07, p-value 0.02)), Additionally, self-reported patient activation or self-rated mental health status did not differ between low and high SES groups after adjusting for confounding variables, respectively (Table 2; β −3.50, p-value 0.25; (−1.54, p-value 0.32).

## 4. Discussion

In this randomized clinical trial of post-stroke older adults who were prescribed smartwatches for AF detection, we found that individuals in the low-income group were more likely associated with anxiety and low self-rated physical health at the end of the study. Studies in in this area have reported that using a wearable has had a positive experience, being a source of multiple psychological benefits and few negative psychological implications for users [8]. To date, there have been no reports of anxiety among low-income group individuals using a smartwatch for AF monitoring.

Previous studies have shown that individuals in low-income groups are more likely to have increased rates of anxiety than those in the high-income group, which is consistent with our findings [17]. One study demonstrated a strong inverse relationship between individuals making an income of $17,500 annually and reporting anxiety disorders (OR 2.6, 95% CI:1.5-5.5, p-value <0.001) [18]. Moreover, a cross-sectional study was conducted in China, where 327 stroke survivors were assessed for anxiety one month after the stroke, and found a significant correlation between anxiety and the low-income group (OR 3.98, 95% CI: 1.60-9.88, p-value 0.003) [19]. Similarly, our study showed that participants in low-income group compared to those in high-income group reported higher anxiety. This finding suggests that income could play a crucial role in an individual’s psychological health, which could potentially affect their physical health, activation, and willingness to manage their overall well-being. When prescribing smartwatches for AF detection in low-income individuals, the healthcare provider should be cognizant of the potential increase in anxiety associated with smartwatch AF monitoring and or any underlying pre-existing anxiety. There could be many underlying aspects causing anxiety in smartwatch users, and further investigation should be done to examine whether improving annual income would decrease underlying anxiety in smartwatch users.

Furthermore, individuals in the low-income group were associated with worse self-reported physical health, a finding that is in agreement with previously reported studies [20]. Ma et al. [21] examined the impact of SES on self-rated health reported that individual economic conditions are strongly associated with self-reported physical health [21]. In another study, including 251 ischemic heart disease patients in Pakistan, Suhail et al. [22] reported that patients in the low-income group had a lower SF-12 PCS score vs. the high-income group patients [22]. Although our findings about the association between SES and physical health are similar to prior reported investigations, our results highlight the importance of SES and self-reported physical health in post-stroke individuals who were prescribed a smartwatch for AF monitoring.

We did not find any association between low-income group individuals and patient activation in post-stroke survivors who were prescribed a smartwatch compared to their counterparts in the high-income groups. This finding was not statistically significant after we adjusted for the confounders, including baseline anxiety score, history of renal disease, and use of statins, as shown in Table 2. Our findings are consistent with a study of a small sample size of 123 patients with AF at the Mayo Clinic. McCabe et al. [23] found no statistically significant difference in patient activation among income levels [23]. Kirkland E. et al. [24] reported that participants with an annual household income of <$20K were less likely to be engaged in monitoring their health than their higher-income peers [24]. It could be possible that low-income individuals have decreased perception of their healthcare condition because of economic instability and everyday struggles; hence, they are not actively involved in managing their health [25]. A more intensive approach may be needed to actively engage low-income individuals prescribed smartwatches for AF monitoring.

The strength of the Pulsewatch study is the ability to monitor AF in elderly stroke survivors with different SES. Standardized, validated tools for assessing patient-reported psychosocial factors were used, enhancing the generalizability and effectiveness of our findings. However, some limitations must be considered while interpreting our study findings. This is a single-center study in which participants were primarily White with tertiary education. The majority of our patients (65.3%) fell into the higher income group. Furthermore, the 44-day period we monitored participants in relation to the smartwatch intervention may not be sufficient to evaluate long-term effects of smartwatch prescription for AF detection in different SES groups.

While our study did control for several variables, including baseline anxiety score, history of renal disease, and the use of statin medication, and utilized standardized questionnaires, we acknowledge that there are important confounders not addressed by our analysis e.g. employment status, disability status that could further influence the outcomes observed. Therefore, we must be cautious in interpreting our findings in the absence of data to directly address these confounders.

Our research suggests that lower socioeconomic status is linked to poorer self-reported health and greater anxiety in stroke survivors using smartwatches for AF detection. Findings underscore the digital divide among individuals from varying SES groups, and the challenges to overcome when integrating technology into clinical settings. Further investigations in a larger cohort to validate our observation and to examine other factors contributing to increasing anxiety and reducing self-reporting of physical health symptoms in individuals from low-income groups are needed.

## 5. Conclusions

Stroke survivors with low baseline income had increased anxiety and reduced self-reporting of physical health over 44 days. Our findings suggest that baseline income may influence psychosocial factors in smartwatch users. Further studies are needed to evaluate how the prescription of a smartwatch affects mood and engagement among post-stroke survivors from diverse SES backgrounds.

## 6. Disclosures

The Pulsewatch Study is funded by R01HL137734 from the National Heart, Lung, and Blood Institute. Dr. Ding’s time is supported by F30HL149335 from the National Heart, Lung, and Blood Institute. Dr. Mehawej’s time is supported by NIH grant 2T32HL120823 from the National Heart, Lung, and Blood Institute. Dr. Tran’s time is supported by K23HL161432 from the National Heart, Lung, and Blood Institute. Dr. Chon’s time was supported by R01 HL137734 and Dr McManus’s time is supported by R01HL126911, R01HL137734, R01HL137794, R01HL135219, R01HL136660, U54HL143541, and 1U01HL146382 from the National Heart, Lung, and Blood Institute. DDM has received honorary, speaking/consulting fee or grants from Flexcon, Rose Consulting, Bristol-Myers Squibb, Pfizer, Boston Biomedical Associates, Samsung, Phillips, Mobile Sense, CareEvolution, Flexcon Boehringer Ingelheim, Biotronik, Otsuka Pharmaceuticals, and Sanofi. Dr. McManus also declares financial support for serving on the Steering Committee for the GUARD-AF study (NCT04126486) and Advisory Committee for the Fitbit Heart Study (NCT04176926).

## Figures and Tables

**Table 1:** Baseline Characteristics according to self-Reported annual baseline income.

Characteristics	Total (N=95)	Income <$50K/yr. (n=33, 34.7%)	Income ≥$50K/yr. (n=62, 65.3%)	p-value
Age, mean, years (SD)	64.9 (9.1)	66 (9.6)	64.5 (8.6)	0.46
Male sex (%)	55 (57.9)	18 (54.5)	37 (59.7)	0.63
Race- Non-Hispanic White (%)	85 (89.5)	30 (90.9)	55 (88.7)	0.194
**Past medical history (%)**				
Congestive Heart Failure	6 (6.3)	1 (3.0)	5 (8.1)	0.337
Cardiac arrhythmias	15 (15.8)	4 (12.1)	11 (17.7)	0.475
Valvular disease	10 (10.5)	4 (12.1)	6 (9.7)	0.712
Vascular disease	25 (26.3)	11 (33.3)	14 (22.6)	0.257
Essential Hypertension	71(74.7)	26 (78.8)	45 (72.6)	0.507
Diabetes	22 (23.2)	6 (18.2)	16 (25.9)	0.402
Hyperlipidemia	81 (85.3)	29 (87.9)	52 (83.9)	0.6
COPD	9 (9.5)	5 (15.2)	4 (6.5)	0.17
Renal disease	5 (5.3)	4 (12.1)	1 (1.6)	**0.029**
Major bleeding event	5 (5.3)	3 (9.1)	2 (3.2)	0.223
Prior myocardial infarction	18 (18.9)	6 (18.2)	12 (19.6)	0.889
Obstructive Sleep Apnea	27 (28.4)	7 (21.2)	20 (32.3)	0.256
**Medication Use (%)**				
Anti-arrhythmic medication	2 (2.1)	2 (6.1)	0 (0.0)	0.05
Beta blocker	40 (42.1)	12 (36.4)	28 (45.2)	0.408
Calcium channel blocker	18 (19)	4 (12.2)	14 (22.6)	0.216
Hypertension medication	53 (55.8)	20 (60.6)	33 (53.2)	0.49
Antiplatelet medication	83 (87.4)	27 (81.8)	56 (90.3)	0.235
Anticoagulant	11 (11.6)	3 (9.1)	8 (12.9)	0.58
Statin	88 (92.6)	28 (84.9)	60 (96.8)	**0.034**
**Physiologic Parameters**				
BMI, mean, Kg/m^2^ (SD)	31.5 ± 19.6	28.3 ± 3.4	33.7 ± 25.2	0.226
Systolic BP, mean, mmHg (SD)	130.7 ± 16.6	135.5 ± 20.1	129.2 ± 14.4	0.082
Diastolic BP, mean, mmHg (SD)	76.0 ± 8.9	77.8 ± 7.8	75.6 ± 9.4	0.259
Heart rate, mean, bpm (SD)	73.6 ± 14.1	75.2 ± 12.8	72.1 ± 15.3	0.331
**Technology Engagement (%)**				
Device Ownership				
Smartphone	81 (85.3)	25 (75.8)	56 (90.3)	0.057
Smartwatch	26 (27.4)	6 (18.2)	20 (32.3)	0.143
**App use frequency**				
Daily	58 (66.0)	16 (55.2)	42 (71.1)	0.461
Never	5 (5.7)	3 (10.3)	2 (3.4)	
Other	25 (28.4)	10 (34.5)	15 (25.4)	
Mean daily wear time (hours)	4.01 (2.3)	3.77 (2.3)	4.16 (2.3)	0.45
**Psychosocial Characteristics (%)**				
Social isolation at baseline	12 (12.6)	6 (18.2)	6 (9.7)	0.235
Cognitive impairment	25 (26.6)	12 (36.4)	13 (21.3)	0.115
**Depression via (PHQ-9)**				
None (Score: 0-4)	51 (54.3)	11 (34.4)	40 (64.5)	**0.004**
Mild (Score: 5-9)	31 (33.0)	12 (37.8)	19 (30.7)	
Moderate (Score: 10-14)	7 (7.5)	4 (12.5)	3 (4.9)	
Moderately severe (Score: 15-19)	4 (4.3)	4 (12.5)	0	
Severe (Score: 20-27)	1 (1.1)	1 (3.1)	0	
Depression via (PHQ-9) (%)	43 (45.7)	21 (65.6)	22 (35.5)	
**Anxiety via GAD-7 score**				
None (Score: 0-4)	64 (68.8)	15 (45.4)	49 (81.7)	**<0.001**
Mild (Score: 5-9)	20 (21.5)	14 (42.4)	6 (10.0)	
Moderate (Score: 10-14)	7 (7.5)	2 (6.1)	5 (8.3)	
Severe (Score: 15 +)	2 (2.2)	2 (6.1)	0 (0)	
Anxiety via GAD-7 score (%)	29 (31.2)	18 (54.5)	11 (18.3)	
**Patient activation (CHAI score)**				
Low (0-79)	31 (34.1)	15 (46.9)	16 (27.1)	0.122
Medium (80-94)	45 (49.5)	14 (43.8)	31 (52.5)	
High (95-100)	15 (16.5)	3 (9.3)	12 (20.4)	

Abbreviations (alphabetically): CHAI: Consumer Health Activation Index; COPD: Chronic Obstructive Pulmonary Disease; GAD-7: Generalized Anxiety Disorder-7; PHQ-9: Patient Health Questionnaire-9; SD: Standard Deviation.

**Table 2:** Linear regression assessing psychosocial outcomes associated with low income group.

	GAD score*			CHAI score**			SF-12 PCS**			SF-12 MCS**		
**Low income (<$50K) vs High income (≥50K)**	**Unadjusted models**											
	Estimate	SE	p-value	Estimate	SE	p-value	Estimate	SE	p-value	Estimate	SE	p-value
	**−2.60**	**0.72**	**0.0004**	**−7.83**	**2.77**	**0.005**	**−7.10**	**1.88**	**0.0002**	**−4.36**	**1.52**	**0.005**
	**Adjusted models**											
	Estimate	SE	p-value	Estimate	SE	p-value	Estimate	SE	p-value	Estimate	SE	p-value
	**2.75**	**0.74**	**0.0003**	−3.50	3.04	0.25	**−5.07**	**2.19**	**0.02**	−1.54	1.55	0.32

* Adjusted for baseline significant variables including history of renal disease, and use of statin medication

** Adjusted for baseline significant variables including baseline anxiety score, history of renal disease, and use of statin medication.
